# The Effect of *Mir-451* Upregulation on Erythroid Lineage Differentiation of Murine Embryonic Stem Cells

**DOI:** 10.22074/cellj.2016.4311

**Published:** 2016-05-30

**Authors:** Narges Obeidi, Ali Akbar Pourfathollah, Masoud Soleimani, Mahin Nikougoftar Zarif, Fatemeh Kouhkan

**Affiliations:** 1 Blood Transfusion Research Center, High Institute for Education and Research in Transfusion Medicine, Tehran, Iran; 2 Department of Hematology, School of Para Medicine, Bushehr University of Medical Sciences, Bushehr, Iran; 3 Department of Immunology, School of Medicine, Tarbiat Modares University, Tehran, Iran; 4 Department of Hematology, School of Medicine, Tarbiat Modares University, Tehran, Iran; 5 Stem Cell Technology Research Center, Tehran, Iran

**Keywords:** MicroRNAs, *Mir-451*, mESCs, Erythropoiesis, Globin Chains

## Abstract

**Objective:**

MicroRNAs (miRNAs) are small endogenous non-coding regulatory RNAs that control mRNAs post-transcriptionally. Several mouse stem cells miRNAs are cloned differentially regulated in different hematopoietic lineages, suggesting their possible role in hematopoietic lineage differentiation. Recent studies have shown that specific miRNAs such as *Mir-451* have key roles in erythropoiesis.

**Materials and Methods:**

In this experimental study, murine embryonic stem cells (mESCs) were infected with lentiviruses containing *pCDH-Mir-451*. Erythroid differentiation was assessed based on the expression level of transcriptional factors (*Gata-1, Klf-1, Epor*) and hemoglobin chains (α, β, γ , ε and ζ) genes using quantitative reverse transcriptase-polymerase chain reaction (qRT-PCR) and presence of erythroid surface antigens (TER-119 and CD235a) using flow cytometery. Colony-forming unit (CFU) assay was also on days 14thand 21thafter transduction.

**Results:**

Mature *Mir-451* expression level increased by 3.434-fold relative to the untreated mESCs on day 4 after transduction (P<0.001). *Mir-451* up-regulation correlated with the induction of transcriptional factor (*Gata-1, Klf-1, Epor*) and hemoglobin chain (α, β, γ, ε and ζ) genes in mESCs (P<0.001) and also showed a strong correlation with presence of CD235a and Ter- 119 markers in these cells (13.084and 13.327-fold increse, respectively) (P<0.05). Moreover, mESCs treated with pCDH-*Mir-451* showed a significant raise in CFU-erythroid (CFU-E) colonies (5.2-fold) compared with untreated control group (P<0.05).

**Conclusion:**

Our results showed that *Mir-451* up-regulation strongly induces erythroid differentiation and maturation of mESCs. Overexpression of *Mir-451* may have the potential to produce artificial red blood cells (RBCs) without the presence of any stimulatory cytokines.

## Introduction

Embryonic stem cells (ESCs) are multipotent stem cells derived from the inner cell mass of the blastocyst, an early-stage embryo (1-3). ESCs keep pluripotency and self-renewing ability due to both their inherent properties and the culture conditions in which they are grown (2). The significance of ESCs in modern biology and medicine derives from two unique characteristics that differentiate them from all other organ-specific stem cells identified to date. First, they can be maintained and enlarged as pure populations of undifferentiated cells for extended periods of time, possibly indefinitely, in culture (3). Secondly, they show a remarkable capacity to form differentiated cell types in culture (4). A close relationship between microRNAs (miRNAs) and ESC development has been observed (5, 6).

Erythropoiesis is the complex process through which a fraction of primitive multipotent hematopoietic stem cells (HSCs) convert to the committed red cell lineage, undergoing differentiation to erythroid progenitors [burst forming unit-erythroid (BFU-E) and colony-forming unit-erythroid (CFU-E)], normoblasts, erythroblasts, reticulocytes, and ultimately mature erythrocytes (7). This process is regulated by many factors including erythropoietin, testosterone, estrogen, interleukin-3 (IL-3), granulocyte-macrophage colony-stimulating factor, IL-9, transcriptional networks and miRNAs (8, 9).

MiRNAs are small endogenous non-coding RNA molecules (19 to 25 nts) that regulate gene expression post-transcriptionally (10) and are phylogenetically conserved (5, 11). While some miRNAs are steadily expressed in the whole organism, their expression pattern is often temporal and/or tissue-specific (12-14). MiRNAs can play significant roles in growth by targeting the transcripts of protein-coding genes and suppressing productive translation (15-17). MiRNAs have shown to be involved in many different cellular processes including metabolism, apoptosis, differentiation, and development (15). Many miRNAs are implicated in a variety of developmental and physiological processes (18, 19). Expression profile of miRNAs in the course of hematopoietic development suggested their potential involvement in hematopoietic differentiation regulation (20, 21). The HSCs lead to both myeloid and lymphoid progenitors (21, 22).

MiRNAs may create a regulatory network with cytokines and transcriptional factors to control erythroid lineage commitment and differentiation (23). Of note, *Mir-451* exists in mature circulating red blood cells (24, 25). Any expression changes of *Mir-451* in murine erythroleukemia (MEL) cells promoted or impaired erythrocyte differentiation, respectively (23, 26). Gata Binding Protein 1 (Globin Transcription Factor 1) [*Gata-1*] is a hematopoietic transcription factor essential for the production of erythrocytes, eosinophils, platelets and mast cells (27). *Gata-1* organizes erythropoiesis by inducing and repressing genes involved in cell division, apoptosis, and terminal maturation (28). *Gata-1* indorses erythroid-specific gene expression through binding at regulatory element sites within the promoters of αand *β-globin* and other erythroid-specific genes (29). Erythropoietin receptor (*Epor*) can induce proliferation of cultured chicken, mouse and human erythroid progenitors. Damaged signaling from the *Epor* not only affects stress erythropoiesis, but also causes erythropoiesis defects during normal development (30). Erythroid Kruppel-like factor (Eklf) (a.k.a. Klf1) is a red cellenriched DNA binding protein that cooperates with its cognate 5´-CCMCRCCCN-3´element within target promoters and enhancers. In genetic, biochemical and molecular studies, the role of Klf1 in β-like globin gene regulation has been emphasized since its discovery (31). Klf1 is a key erythroid transcriptional regulator (32, 33) and induces a different set of genes associated with erythropoiesis including the *β-globin* gene (Hbb) (34).

In this experimental study, we examined the early stages of mESCs lineage commitment by investigating whether *Mir-451* up-regulation could induce erythropoiesis differentiation from mESCs and be used as a replacement to the stimulatory cytokines for mESCs differentiation into erythroid cells. 

## Materials and Methods

### HEK-293T cell line culture

Human embryonic kidney (HEK)-293T cell line was obtained from the National Cell Bank of Iran (Pasteur Institute, Iran). The HEK-293T cells were cultured in Dulbecco’s modified Eagle’s medium (DMEM), 10 % fetal bovine serum (FBS), 100 U/ml penicillin, 2 mM L-glutamine and 100 µl streptomycin (all from Gibco, USA). This cell line was kept at 37˚C in a humidified atmosphere containing 95 % humidity and 5 % CO_2_ according to the supplier’s instructions.

### Recombinant lentiviruses production 

The pCDH-451 plasmid was produced by li-gating 250 bp fragments encompassing *pri-Mir-451* sequences into the XbaI /BamHI restriction sites of the pCDH-CMV-MCS-EF1-copGFP vector (System Biosciences, USA). These fragments were elevated by polymerase chain reaction (PCR) reaction using following primers: *pri-Mir-451* F: 5′-GTC GTA TGC AGA GCA GGG TCC GAGGTA TTC GCA CTG CAT ACG ACA ACT CA3′ and R: 5′GTCGTATGCAGAGCAGGGTCCGAGGTATTCGCACTGCATACGACAACCTC-3′ on extracted genomic DNA. For lentivirus production; HEK-293T cells (3×10^3^) were seeded into 10-cm plates containing DMEM medium supplemented with 10% FBS. The day after, pPAX2 plasmid (containing gag and pol genes) and pMD2 plasmid (containing vsv gene) were co-transfected with the pCDH-451 plasmid empty vector (pCDH empty backbone) as negative control into seeded HEK-293T cells using the lipofectamin 2000 reagent (Invitrogen, USA) according to the manufacturer’s protocol. The supernatants containing generated lentiviruses were collected every 12 hours for 3 days after transfection and concentrated by ultracentrifugation at 40.000 g for 2 hours. Then for virus titration, HEK-293T cells were transduced with a different concen- tration of recombinant lentiviruses and the number of viruses in the functional copy was detected using green fluorescent protein (GFP) protein and fluorescent microscope forty-eight hours later.

### Murine embryonic stem cells culture 

Murine ESC (mESC) [E14Tg2A] lines were cultured on gelatin-coated tissue culture dishes (Sigma, USA) at an intensity of 40,000 cells/cm^2^ . ESC medium, which was exchanged daily, contained knockout DMEM, 20% FBS-ES,1 mM sodium pyruvate (Gibco, USA), 2 mM Glutamine (Euroclone, Italy), 0.05 mM b-mercaptoethanol, 1 mM non-essential amino acids (Gibco, USA), 1,000 U/ml recombinant mouse leukemia inhibitory factor (LIF, Sigma, USA) and 100 U/ml penicillin/streptomycin (Euroclone, Italy).

### Murine embryonic stem cells infection 

The infection was done in three groups. Each groups had three samples. Embryonic bodies (EB) were cultured for 1 to 21 days under the following conditions: i. Blank: EBs did not receive any treatment (untreated group), ii. pCDH-451 lentiviruses: EBs were transduced with pCDH-451 lentiviruses (pCDH-451 group) and iii. pCDH-empty lentiviruses: EBs were transduced with pCDH-empty lentiviruses (negative control group).

After 14 and 21 days, the effect of *Mir-451* upregulation in erythroid differentiation was monitored by analyzing expression of transcriptional factor (*Gata-1, Klf-1* and *Epor*) and hemoglobin chain (α, β, γ, ε and ζ) using quantitive reverse transcriptasePCR (qRT-PCR) and presence of erythroid cell surface markers (CD235a and Ter-119) using flow cytometry.

### RNA extraction 

Total RNA was extracted from test and control groups using the Trizol reagent (Gibco, USA), according to the manufacturer’s instructions. cDNA was synthesized by Superscript II reverse transcription (Invitrogen, USA) and random hexamer primers, according to the manufacturer’s instructions.

### Real-time reverse transcriptase-polymerase chain reaction quantification of miRNAs 

Real-time RT-PCR quantification of miRNAs was undertaken using primers designed Primer Express version 2.0 (Applied Biosystems, Foster City, CA). Briefly, first cDNA strand was synthesized through miRNA 1st-strand cDNA synthesis kit (Stratagene, USA) and reverse transcribed into qPCR-ready cDNA. After that, miRNA qRT-PCR was carried out in triplicate on ABI PRISM 7500 real time PCR System (Applied Biosystems, USA) with the high-specifity miRNA qPCR core reagent kit (Stratagene, USA) and normalized to U6 small nuclear RNA (Snord47) as an endogenous control. Primer sequences are shown in Table 1. The qRT- PCR cycling conditions were 10 minutes at 95˚C followed by 40 cycles of 10 seconds at 95˚C, 15 seconds at 60˚C, and 20 seconds at 72˚C. Data analyses were performed using the 2^−ΔΔct^ method.

**Table 1:** The sequence of primers that used in this study


Gene	Primer (5'-3')

*Mir-451*	F: CGA GAA ACC GTT ACC ATT AC
	R: GAG CAG GGT CCG AGG T
*Snord47*	F: ATC ACT GTA AAA CCG TTC CA
	R: GAG CAG GGT CCG AGG T
* Gata-1*	F: CAC TCC CCA GTC TTT CAG G
	R: TGC CGT CTT GCC ATA GG
* Klf-1*	F: CGC ACA CGG GAG AGA AG
	R: ACA GCA GAA GGG ACG ATG
*Epor*	F: ATA TCA ATG AAG TAG TGC TCC TG
	R: CCC TTT GTG TCC CTC CTG
* ζ chain*	F: CAA CTT CAA GCT CCT GTC C
	R: GGA GGG TTC AAT AAA GGG
* ε chain*	F: GGG AAG GCT CCT GAT TG
	R: CAC TGA GAT GAG CAA AGG TC
*γ chain*	F: AAC TTC AAA CTC TTG GGT AAT G
	R: GGA GGC ATA GCG GAC AC
*β chain*	F: CTG ATT CTG TTG TGT TGA CTT G
	R: GAC AAC CAG CAG CCT GC
*α chain*	F: CTG GAA AGG ATG TTT GCT AG
	R: CAT CGG CGA CCT TCT TG
*β.actin*	F: CTT CTT GGG TAT GGA ATC CTG
	R: GTG TTG GCA TAG AGG TCT TTA C


### Real-time reverse transcriptase-polymerase chain reaction quantification of transcriptional factors and hemoglobin chains 

Expression of transcriptional factor (*Gata-1, Klf- 1, Epor*) and hemoglobin chain (α, β, γ, ε and ζ) genes was quantified using ABI PRISM 7500 real-time PCR System (Applied Biosystems, USA) with the SYBR premix ExTaq kit (Takara, Japan) according to the manufacturer’s instruction. The qRT-PCR cycling conditions were done same as above.

### Flow cytometry 

Cells from all groups (blank control group, pCDH-451 group and negative control group) were collected for flow cytometry. The viability of the cells was examined by trypan blue exclusion and was always greater than 95%. They were immunostained with phycoerythrin (PE)–conjugated anti-TER119 (1:200) and PE–conjugated anti-CD235a (1:200, BD Pharmingen, San Diego, CA, USA) antibodies. Propidium iodide was added to exclude dead cells from analysis. The cells were then analyzed on flow cytometer PartecPAS III (Partec, Germany).

### Colony-forming unit assays 

Colony-forming cell (CFC) assay was carried out in triplicate using methylcellulose complete media (MethoCultTM, StemCell Technologies, Inc, USA) according to the manufacturer’s instructions. In all groups, 5×10^3^ mESCs were cultured in 35-mm plates with the medium containing 25% FBS, 2% bovine serum albumin, 1.3% methyl cellulose, 0.05 mmol/L 2-mercaptoethanol, 3 U/mL erythropoietin (EPO), 2 mmol/L L-glutamine, 50 ng/mL stem cell factor (SCF), 10 ng/mL IL-3 and 10 ng/mL granulocyte macrophage-colony-stimulating factor plus activin A (25 ng/mL). After incubation at 37˚C, 5% CO_2_ and 95% humidity for 12 days, the colony-forming unit-granulocyte, erythroid, macrophage, megakaryocyte (CFU-GEMMs), colony-forming unit-granulocyte, macrophage (CFU-GMs) and CFU-Es in every dishes was sorted and counted under a high-quality inverted microscope (Leica, Heidelberg, Germany).

### Statistical analysis 

All tests were repeated three times and data were shown as mean ± SD. The comparison between groups was performed using the Student’s t test. P value less than 0.05 was considered statistically significant.

## Results

### Transfection efficiency and production of lentiviruses in HEK293T cells

The pMD2G, psPAX2 and pCDH-451 plasmids were co-transfected into HEK293T cells on a 10 cm plate using lipofectamin 2000 reagent (Invitrogen, USA) according to the manufacturer’s protocol (pCDH-451). Lentiviruses expressing *Mir-451* was then generated.

Lentiviral vectors created from pCDH-empty plasmids were used as negative control (pCDH- Neg). Transfection efficiency was confirmed each time by fluorescent microscopy. Approximately 95% of cells in the pCDH-451 group and 97% of cells in the pCDH-empty vector group with green fluorescence were distinguished 48 and 72 hours after infection (Figes.1, 2). No fluorescent-positive cell was present in our control group.

### Transduction efficiency and Mir-451 expression in murine embryonic stem cells 

In order to enter mESCs into erythroid commitment, mESCs were transduced with lentiviral vector pCDH-451 expressing copGFP and allowed to form EBs in suspension culture. CopGFP serves as an internal control by marking all cells that receive the vector. The concentrations of this vector was in the range of 3×10^7^ to 7×10^7^ viral particles per milliliter and diverse multiplicities of infection were used to optimize transduction conditions. Transduction efficiency was monitored each time by fluorescent microscopy and evaluated by flow cytometry for the GFP marker. GFP overexpression of lentiviruses in mESCs was 60% of cells with pCDH-451 and 65% of cells with pCDH-empty vectors and was distinguishable 96 hours after infection. No fluorescent-positive cell was detected in our control group (Fig.3). 

**Fig.1:**
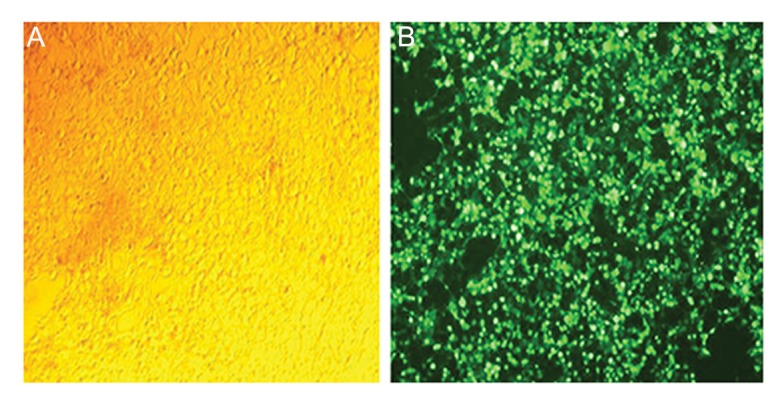
A. Transfected HEK293T cells examined by light microscopy and B. Transfected HEK293T cells examined by fluorescent microscopy. Transfection efficiency of murine embryonic stem cells (mESCs) with *pCDH-Mir-451* was more than 95% as determined by fluorescent microscopy.

**Fig.2:**
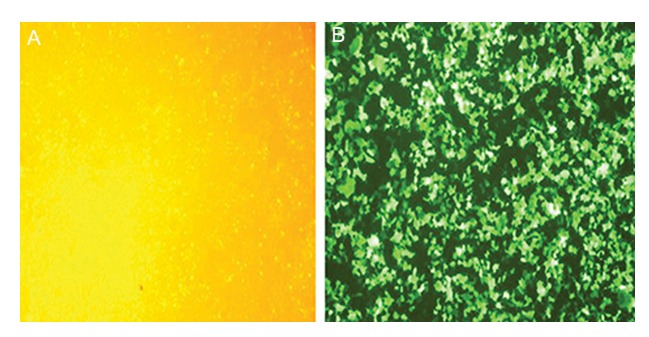
A. Transfected HEK293T cells examined by light microscopy and B. Transfected HEK293T cells examined by fluorescent microscopy. Transfection efficiency of murine embryonic stem cells (mESCs) with pCDH-empty vector was more than 95% as determined by fluores- cent microscopy.

**Fig.3:**
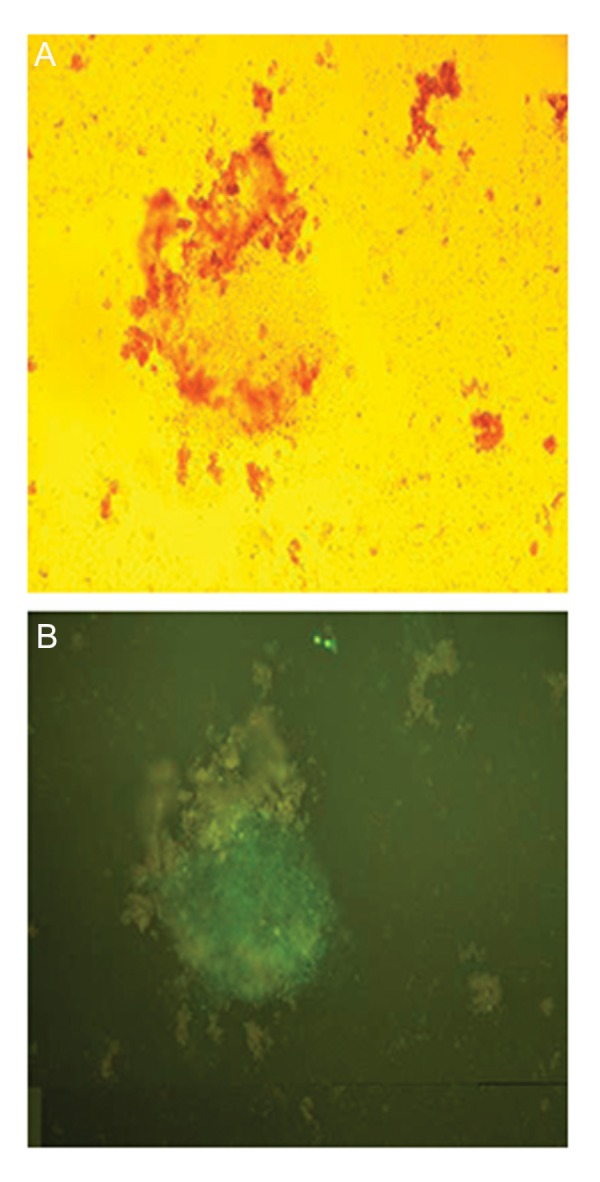
A. Transduced murine embryonic stem cells (mESCs) examined by light microscopy and B. Transduced mESCs examined by fluorescent microscopy. Transduction efficiency of mESCs with pCDH- *Mir-451* was more than 60% as determined by flow cytometry for the GFP marker.

### Recombinant lentiviruses increased mature miRNAs level in treated murine embryonic stem cells 

**Fig.4:**
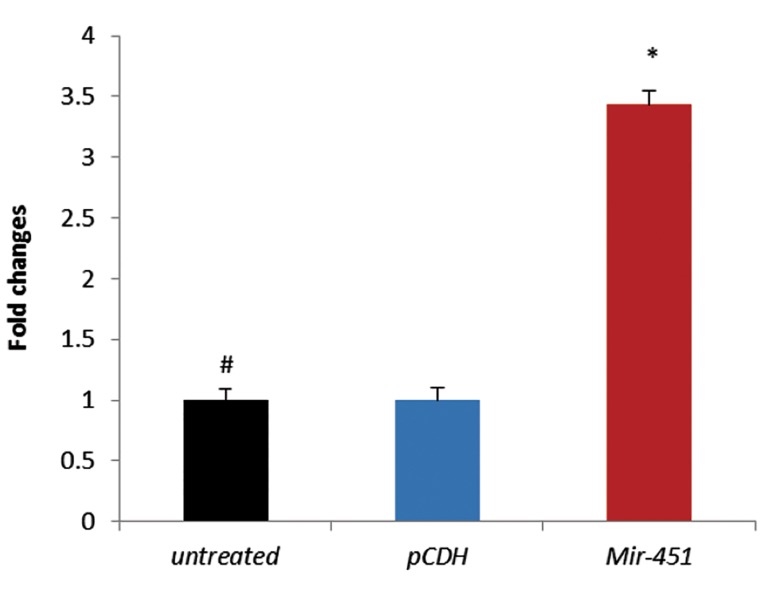
Expression analysis (fold changes) of *Mir-451* in treated murine embryonic stem cells (mESCs) on day 4. The expression of *Mir-451* in the mESCs treated with *pCDH-Mir-451* cells was significantly higher than that in mESCs treated with pCDH-empty vector and those untreated. Relative miRNA expression levels were normalized to Snord47 as an internal control. Columns, mean of three different experiments. *; P<0.001 and #; Results were compared with these columns.

We determined the expression level of *Mir-451* on day 4 after transduction in test and control groups by qRT-PCR. In mESCs treated with pCDH-*Mir-451* lentiviruses, mature *Mir-451* expression level increased by 3.434-fold relative to the untreated mESCs (P<0.001, Fig.4). As expected, when mESCs were treated with pCDH-empty lentiviruses, mature Mir- 451 expression level displayed no significant alteration compared with blank control groups (P>0.05). These results suggested that *pCDH-Mir-451* recombinant lentiviruses are efficient and increased mature *Mir-451* level significantly. 

### Expression analysis of transcriptional factor genes 

**Fig.5:**
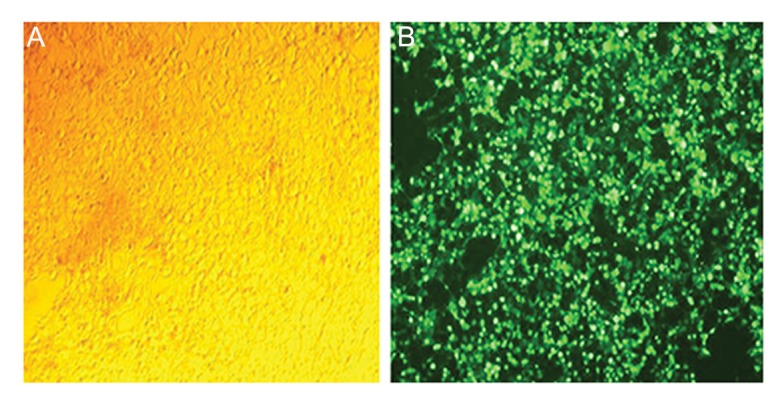
A. Expression analysis (fold changes) of transcriptional factors in treated murine embryonic stem cells (mESCs) on day 14 and B. Expression analysis (fold changes) of transcriptional factors in treated mESCs on day 21. Relative transcriptional factors expression levels were normalized to β-Actin as an internal control. Results presented as fold change compared with the control group. Columns, mean of three different experiments. *; P<0.001 and #; Results were compared with these columns.

We then examined the effect of this up-regulation on the expression of erythroid specific markers (*Gata-1, Klf-1* and *Epor*) by qRT-PCR on day 14 and 21, as an index of erythropoiesis. According to qRT-PCR results, the expression of these transcriptional factors distinguished the pCDH-451, indicating successful erythropoeisis (Fig.5). In the mESCs treated with *pCDH-Mir-451*, *Gata-1* and *Klf-1* expression were increased by 1.952and 4.084-fold, respectively when compared with the untreated control group on day 14 (P<0.001) but was decreased by 0.712and 2.454-fold, respectively on day 21 (P<0.001). In this group, *Epor* expression was increased on day 14 and 21. Treatment of mESCs with *pCDH-Mir-451* lentiviruses, led to the rise of *Epor* expression by 23.183-fold relative to the untreated mESCs on day 21 (P<0.001).

### Expression analysis of hemoglobin chain genes

**Fig.6:**
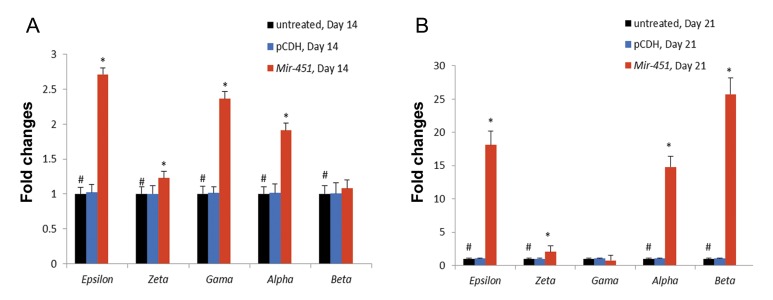
A. Expression analysis (fold changes) of hemoglobin chains in treated murine embryonic stem cells (mESCs) on day 14 and B. Ex- pression analysis (fold changes) of hemoglobin chains in treated mESCs on day 21. Relative hemoglobin chains expression levels were normalized to β-Actin as an internal control. Results presented as fold change compared with the control group. Columns, mean of three different experiments. *; P<0.05 and #; Results were compared with these columns.

The expression profile of hemoglobin chain genes was obtained using qRT-PCR method on days 14 and 21. According to the qRT-PCR results, mESCs treated with *pCDH-Mir-451* led to a significant increase of ε, ζ, γ and α transcripts (by 2.824-1.421, 2.566and 1.918-fold, respectively) compared with the untreated control group on day 14 (P<0.05). On day 21, sharp increase of accumulation of ε, α and *β-globin* transcripts were detected in the pCDH-451 group (by 18.126- 14.774and 25.723, fold-respectively) compared with the untreated control group (P<0.05). A moderate increase of ζ transcripts (by 2.035-fold) was seen in mESCs treated with *pCDH-Mir-451* on day 21 (P<0.05). The pattern of γ transcripts was decreased in this group (by 0.742-fold) compared with the untreated control group (P>0.05). These results further confirmed that *Mir-451* may have a vital roles in the induction of hemoglobinization (Fig.6).

### Flow cytometry analysis of TER-119 and CD235a expressions 

As other indicators of erythropoiesis, the presence of TER119 and CD235a was estimated using flow cytometry on days 14 and 21. As shown in Table 2, in the pCDH-451-infected group, overexpression of *Mir-451* led to a rise in the proportion of cells expressing TER119 and CD235a 30.12 ± 2.34% for and 17.47 ± 2.21%, respectively, compared with 3.87 ± 0.95% and 2.56 ± 0.87% of the control cells (untreated mESCs), respectively, on day 14 (7.782and 6.824-fold, respectively, P<0.05). Results on day 21 showed the percentage of cells positive for TER119 and CD235a was 66.34 ± 2.81% and 46.38 ± 2.37% in *Mir-451* treated mESCs and 5.07 ± 1.01% and 3.48 ± 1.28% in untreated mESCs, respectively (13.084and 13.327-fold, respectively, P<0.05) (Figes.7, 8). 

### Colony-forming unit assays

On the 12^th^ day after incubation, the cells in the three groups created three types of colonies, indicating their ability to develop different progenitor cells (Fig.9). The number of *Mir-451* in treated mESCs, pCDH-empty vector and untreated mESCs in treated-formed colonies, CFU-E, CFU-GM, and CFU-GEMM colonies are shown in Table 3. According to CFU assay results, mESCs treated with *pCDH-Mir-451* led to a significant increase in CFU-E colonies (by 5.2-fold) compared with the untreated control group (P<0.05).

**Table 2:** The proportion of cells expressing TER119 and CD235a in three mESCs groups by FACS


Groups	Day 14	Day 21

The proportion of the cells expressing TER119		
Treated mESCs with *Mir-451*	30.12 ± 2.34%	66.34 ± 2.81%
Treated mESCs with pCDH-empty	4.02 ± 1.21%	7.90 ± 1.41%
Untreated mESCs	3.87 ± 0.95%	5.07 ± 1.01%
The proportion of the cells expressing CD235a		
Treated mESCs with *Mir-451*	17.47 ± 2.21%	46.38 ± 2.37%
Treated mESCs with pCDH-empty	2.98 ± 1.36%	6.0 3 ± 1.19%
Untreated mESCs	2.56 ± 0.87%	3.48 ± 1.28%


mESCs; Murine embryonic stem cells.

**Table 3:** Colony-forming ability of *Mir-451* in treated mESCs, pCDH-empty treated mESCs and untreated mESCs


Colonies	CFU-GEMM	CFU-GM	CFU-E
Groups			

*Mir-451* in treated mESCs	20 ± 2.34	12 ± 2.81	26 ± 2.37
pCDH-empty in treated mESCs	1 8 ± 1.21	17 ± 1.41	6 ± 1.36
Untreated mESCs	17 ± 1.01	18 ± 1.19	5 ± 1.28


mESCs; Murine embryonic stem cells, CFU-GEMM; Colony-forming unit-granulocyte, erythroid, macrophage,

**Fig.7:**
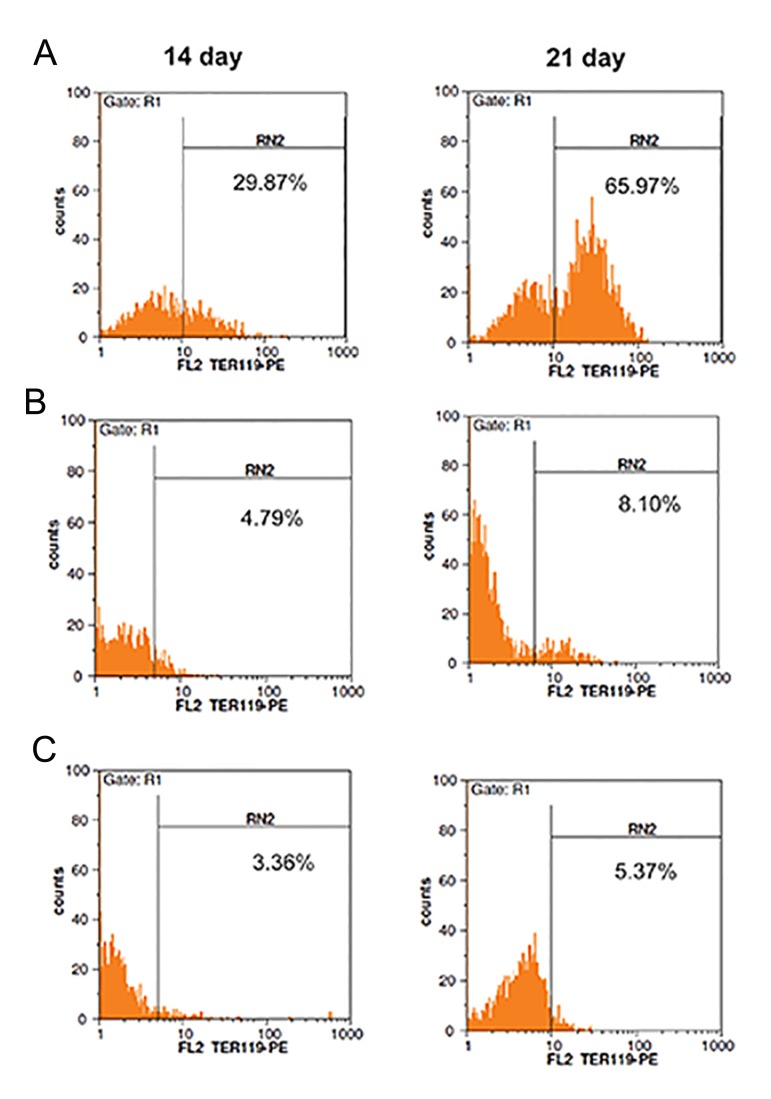
Overexpression of TER-119 in murine embryonic stem cells (mESCs). More than 95% of cells were gated in R1. A. FACS histogram showing transduction efficiency of mESCs with lentiviral vector expressing *pCDH-Mir-451*, B. FACS histogram showing transduction efficiency of mESCs with lentiviral vector expressing pCDH-empty vector and C. FACS histogram showing transduction efficiency of untreated mESCs. The positive regions were adjusted according to the control isotope antibody reaction.

**Fig.8:**
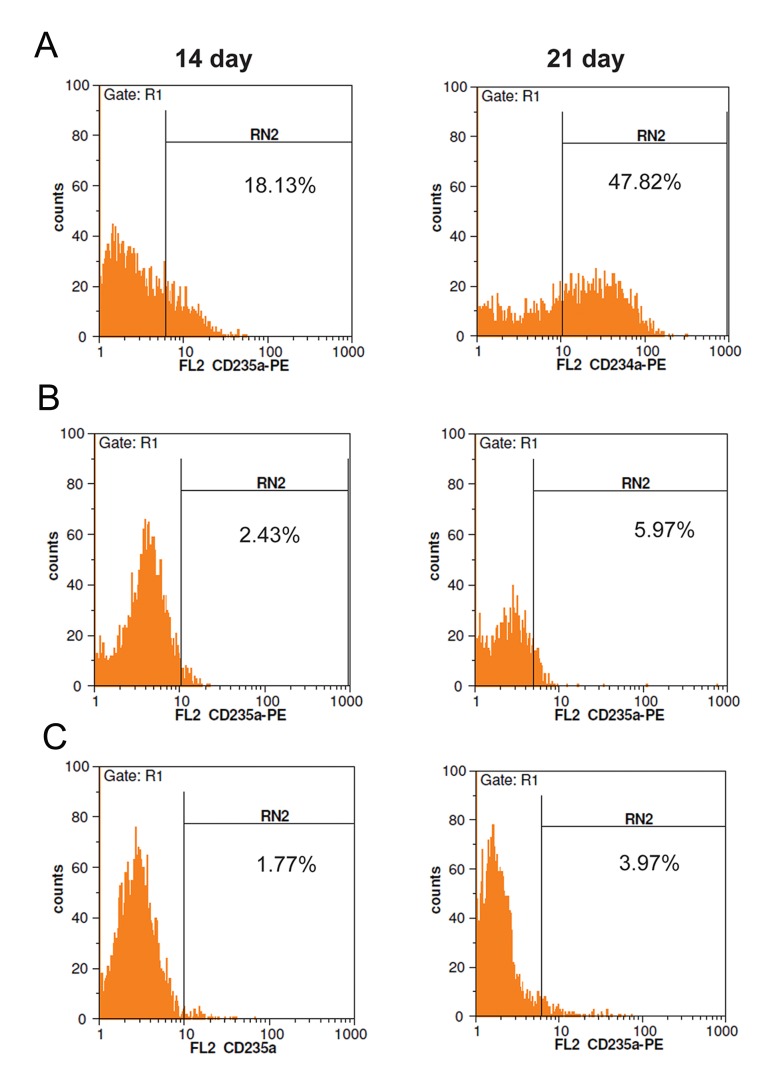
Overexpression of CD235a in murine embryonic stem cells (mESCs). More than 95% of cells were gated in R1. A. FACS histogram showing transduction efficiency of mESCs with lentiviral vector expressing *pCDH-Mir-451*, B. FACS histogram showing transduction efficiency of mESCs with lentiviral vector expressing pCDH-empty vector and C. FACS histogram showing transduction efficiency of untreated mESCs. The positive regions were adjusted according to the con- trol isotope antibody reaction.

**Fig.9:**
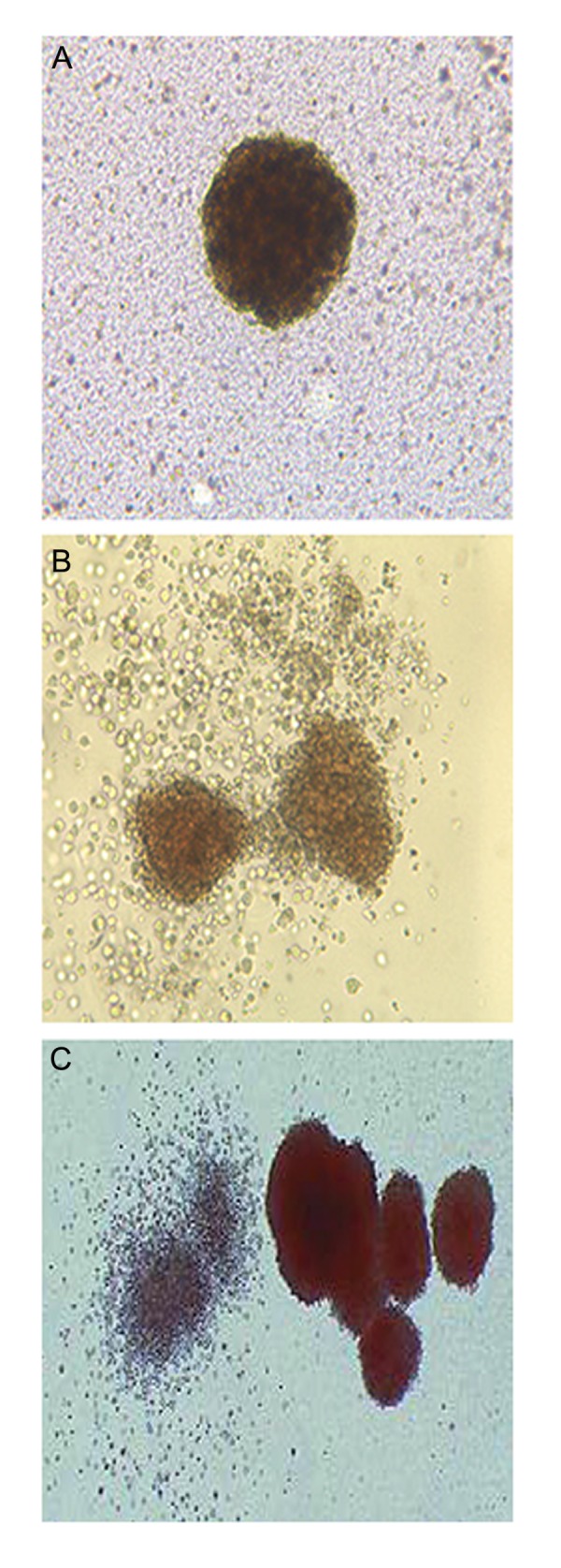
CFC assay of murine embryonic stem cells (mESCs), A. CFU-E, B. CFU-GEMM and C. CFU-GM with some CFU-E, all observed under an inverted microscope (×100) showing that mESCs generated the colonies. CFU-E; Colony-forming unit-erythroid, CFU-GEMM; CFU- granulocyte, erythroid, macrophage, megakaryocyte and CFU-GM; CFU-granulocyte, macrophage.

## Discussion

Erythropoiesis requires the regulation of several pathways to enable the production of vast numbers of red blood cells (RBCs) over a person’s lifetime (35,36). The particular biological functions of individual miRNAs are now appearing through reverse genetic studies, revealing important roles in development, physiology and disease, including hematopoiesis (19,37). MiRNAs play important roles in regulation of a multitude of physiological functions, such as stem cell differentiation and development. Precise regulation of these processes is vital to normal development and prevention of cancer. The aim of some large studies was to identify the roles of miRNAs in differentiation in different organs (38,40) including hematopoietic lineage differentiation (41,42). MiRNAs, because of their small size, nuclease resistance, fast synthesis and long half-life/bioactivity may be the ideal substitutes for growth factors for direct differentiation towards any particular cell type (43). Several murine miRNA loci have recently been disturbed by gene targeting with resultant hematopoietic phenotypes (e.g., mice lacking *Mir-155*, a lymphoid-restricted miRNA, have defective immune responses) (44). In this study, we found a new protocol to differentiate mESCs into erythroid lineage by expression modulation of specific miRNAs in the absence of any erythroid-specific cytokines. mESCs were treated with pCDH-451 lentiviruses and the emergence of erythroid lineage was investigated. In our EB differentiation system, overexpression of *Mir-451* in mESCs induced the differentiation of erythroid cells. Our observation seems to be in agreement with a previous study by Kouhkan et al. (45) who demonstrated that *Mir-451* have a strong positive correlation with the appearance of erythroid specific cell surface markers such as CD71 and CD235a, and hemoglobin synthesis upon erythroid differentiation of CD133+ cells and Pase et al. (26) also showed that *Mir-451* accelerated the rate of erythrocyte maturation, an action mediated in part by repression of gata2.In addition, they showed that *Mir-451* is significantly up-regulated during erythroid differentiation. *Mir-451* plays an important role in promoting erythroid maturation, in part via its target GATA-2. 

As markers of erythropoiesis, we examined the expression of *Gata-1, Epor*, and *Klf-1* transcription factors using qRT-PCR in all groups. Results revealed that these factors were expressed in mESCs transduced with lentiviral vector expressing pCDH-*Mir-451*. *Gata-1* expression was decreased in all groups on day 21. *Gata-1* reveals physiologically that occur during normal erythropoiesis (46). During transcriptional effects or physical interactions with core cell cycle components, *Gata-1* could obstruct cell proliferation (47). Rylski et al. (47) showed that *Gata-1* persuades G1 arrest during erythroid maturation and identified an extensive *Gata-1*-regulated network of gene activation and repression related to cell cycle control. *Epor* expression was increased in the pCDH-451 group on day 21. Erythropoietin (Epo) is a glycoprotein and a major regulator of the growth and differentiation of erythroid blood cells. Its biological influence is mediated through binding to the *Epor* on the cell surface (48). Klf1 expression was decreased in all groups on day 21. Cantor and Orkin (49) have shown that binding sites for both *Klf-1* (and the related ubiquitously expressed protein Sp1) and *Gata-1* are located in close proximity in cis-regulatory elements of erythroid-specific genes. In addition, both Sp1 and *Klf-1* physically associate with the zinc finger region of *Gata-1* and synergistically activate *Gata-1* target genes in transiently expressed reporter constructs. Thus, protein-protein interactions between *Gata-1* and *Klf-1* may be implicated in facilitating the switch from fetal to adult globin expression. 

An additional study on the expression profile of hemoglobin chains using qRT-PCR indicated that the up-regulation of *Mir-451* induced a significant rise in mESC hemoglobinization and similarly we detected a sharp increase of accumulation of *αglobin* and *β-globin* transcripts in the pCDH-451 group. Therefore, *Mir-451* seems to have more effect on the progression of erythroid maturation that increasing expression level of *α-globin* and *β-globin*. These results are consistent with some previous studies indicating that *Mir-451* has a strong positive correlation with the late stage of erythropoiesis (41,42,45,50,51). On the other hands, *Mir-451* stimulated embryonic *globin* chains (ζ and ε) and *γ-globin*. In the first step of erythroid differentiation, expression level of *γ-globin* was at high level and at the late step of it, *γ-globin* expression was low (51). According to our results, *ζ-globin* and *ε-globin* expression were elevated in the pCDH-451 group on day 21. *ζ-globin* is an essential globin chain for embryonic Hb such as Gower I (ζ2ε2), Portland I (ζ2γ2) and Portland II (ζ2β2) (52). In addition, expression level of *Gata-1* was decreased on day 21. Raich et al. (53) showed that *Gata-1* obstruct human epsilon globin transcription by binding to its proximal promoter. In mice, erythropoiesis begins in the embryonic yolk sac where primitive erythroid cells express εy and *bh-1* globins. The εy gene is suppressed in definitive erythroid cells. In definitive erythropoiesis, ε is expressed and suppressed autonomously, however, in primitive erythropoiesis ε seems to be regulated competitively (54,55). 

In this study, we isolated mESCs treated with pCDH-*Mir-451* and confirmed that they display stem cell properties based on CFC assays, consistent with similar findings obtained with HSCs (22,56). We also isolated mESCs treated with pCDH empty vector and untreated mESCs as we control groups to analyze miRNA expression profile. mESCs are a mixed population consisting predominantly, almost 90%, of differentiated, committed hematopoietic progenitor cells (HPCs). We compared miRNA expression profiles of these three mESCs subpopulations to detect differentially expressed miRNAs. 

We examined the effect of overexpression of Mir451 on erythroid differentiation of mESCs. FACS results indicated that *Mir-451* up-regulation induced the erythroid surface markers TER119 and CD235a. CD235a expression increased on day 14 and reached its peak level on day 21. These results were similar to those reported by Choong et al. (57) and Kouhkan et al. (45,51). TER119 expression increased upon erythroid differentiation. Kina et al. (58) demonstrated that TER-119 was highly specific to erythroid cells at the stages from early proerythroblast to mature erythrocyte and that TER-119 recognizes a cell surface molecule which is strongly associated with glycophorin A. It was shown that TER-119 was expressed only on normal erythroid cells but not on erythroleukaemia cells, even after induction of these cells with dimethylsulphoxide (DMSO). 

## Conclusion

We show that *Mir-451* up-regulation may play important roles in erythroid differentiation for *in vitro* erythropoiesis of mESCs and production of artificial RBCs without the presence of any stimulatory cytokines. Since the major problem of patients with hemoglobinopathies, such as sickle cell anemia and thalassemia, is failure in the production of adult globin (HbA) and reactivation of the αand *β-globin* chains has been shown to rescue the lethality of mice with αand β-thalassemia. *Mir-451* and other miRNAs may be useful in designing effective therapeutic strategies for the possibility of reversing these abnormalities by gene therapy. 
